# New Strategies to Fight against Sarcopenia at Old Age

**DOI:** 10.1155/2012/676042

**Published:** 2012-04-26

**Authors:** Dominique Meynial-Denis, Olivier Guérin, Stéphane Michel Schneider, Dorothee Volkert, Cornel Christian Sieber

**Affiliations:** ^1^INRA, UMR 1019, Human Nutrition Unit, Research Center of Human Nutrition (CRNH Auvergne), 63000 Clermont-Ferrand, France; ^2^CHU Nice Pole of Gerontology, CNRS, UMR 6267, INSERM U998, University of Nice Sophia Antipolis, 06202 Nice, France; ^3^Gastroenterology and Clinical Nutrition, CHU of Nice, INSERM U1065, University of Nice Sophia Antipolis, 06200 Nice, France; ^4^Institute for Biomedicine of Aging, University of Erlangen-Nürnberg, 90419 Nürnberg, Germany

The focus of this special issue is to determine new strategies to fight against sarcopenia in old age.

Aging is associated with a progressive loss of muscle mass and strength, a process called sarcopenia. The most evident metabolic explanation for muscle decline in elderly people is an imbalance between protein synthesis and breakdown rates, but other causes, such as neurodegenerative processes, reduction in anabolic hormone production or sensitivity (e.g., insulin, growth, and sex hormones) and in capacity to respond to anabolic stimuli (e.g., amino acids, exercise), dysregulation of cytokine secretion, modifications in the response to inflammatory events, inadequate nutritional intake and sedentary lifestyle, are involved. The sequelae of sarcopenia often contribute to frailty, decreased independence, and subsequently increased health care costs. In this issue, a few aspects of new strategies to fight against sarcopenia are presented, including

- role of disease in sarcopenia,
- new strategies and/or therapeutics in sarcopenia,
- muscle performance and sarcopenia/physical activity and sarcopenia,
- antioxidants and aging /sarcopenia.

In a clinical study, Domanski and Ciechanowski explain how the whole-body protein-energy deficiency, called protein-energy wasting, aggravates sarcopenia in elderly patients suffering from chronic kidney disease.

Novel strategies attenuating sarcopenia are presented in two reviews by Robinson et al. (optimising diet and nutrition throughout life, e.g., sufficient protein intake, vitamin D, and antioxidant nutrients combined with resistance exercise training interventions) and by Sakuma and Yamaguchi (myostatin inhibition, supplementation with eicosapentaenoic acid or ursolic acid, activation of peroxisome proliferator-activated receptor *γ* coactivator-1*α* (PGC-1*α*) by exercise).

Kemmler et al. present a review on the benefits of physical exercise in preventing sarcopenia and related muscle malfunction. They underline the lack of motivation or the physical limitations of elderly subjects and present two new exercise technologies, whole-body vibration and whole-body electromyostimulation, which are also feasible in unmotivated or handicapped persons. However, while these methods increase strength and power parameters, they do not improve muscle mass. In clinical studies, Sayers and Gibson examine whether high-speed power training (HSPT) improves muscle performance and braking speed using a driving stimulator. They demonstrate that HSPT exerts a wider effect and improves speed compared to slow-speed strength training. Marcus et al., in another clinical study, evidence that thigh intramuscular adipose tissue plays a potent role in reducing mobility function and physical activity in older adults.

Cerullo et al. present a review on the benefits of oral antioxidants in the prevention and treatment of sarcopenia. They demonstrate that oral antioxidant supplementation may contribute to reducing indices of oxidative stress in both animal and human models by reinforcing the natural endogenous defences. 

This special issue provides the opportunity to discuss the challenges of sarcopenia and proposes creative solutions to fight against it in old age and to contribute to “aging well.”

*Dominique Meynial-Denis* *Dominique Meynial-Denis* *Olivier Guérin* *Olivier Guérin* *Stéphane Michel Schneider* *Stéphane Michel Schneider* *Dorothee Volkert* *Dorothee Volkert* *Cornel Christian Sieber* *Cornel Christian Sieber*

